# Induction of Glycerol Synthesis and Release in Cultured *Symbiodinium*

**DOI:** 10.1371/journal.pone.0047182

**Published:** 2012-10-11

**Authors:** Luis P. Suescún-Bolívar, Roberto Iglesias-Prieto, Patricia E. Thomé

**Affiliations:** Unidad Académica de Sistemas Arrecifales Puerto Morelos, Instituto de Ciencias del Mar y Limnología, Universidad Nacional Autónoma de México, Mexico City, Mexico; UC Merced, School of Natural Sciences, United States of America

## Abstract

**Background:**

Symbiotic dinoflagellates transfer a substantial amount of their photosynthetic products to their animal hosts. This amount has been estimated to represent up to 90% of the photosynthetically fixed carbon and can satisfy in some instances the full respiratory requirements of the host. Although in several cnidarian-dinoflagellate symbioses glycerol is the primary photosynthetic product translocated to the host, the mechanism for its production and release has not been demonstrated conclusively.

**Principal Findings:**

Using *Symbiodinium* cells in culture we were able to reproduce the synthesis and release of glycerol *in vitro* by employing an inductor for glycerol synthesis, osmotic up-shocks. Photosynthetic parameters and fluorescence analysis of photosystem II showed that the inductive conditions did not have a negative effect on photosynthetic performance, suggesting that the capacity for carbon fixation by the cells was not compromised. The demand for glycerol production required to attain osmotic balance increased the expression of ribulose 1,5-bisphosphate and of glycerol 3-phosphate dehydrogenase, possibly competing with the flux of fixed carbon necessary for protein synthesis. In longer exposures of cultured *Symbiodinium* cells to high osmolarity, the response was analogous to photoacclimation, reducing the excitation pressure over photosystem II, suggesting that *Symbiodinium* cells perceived the stress as an increase in light. The induced synthesis of glycerol resulted in a reduction of growth rates.

**Conclusions:**

Our results favor a hypothetical mechanism of a signaling event involving a pressure sensor that may induce the flux of carbon (glycerol) from the symbiotic algae to the animal host, and strongly suggest that carbon limitation may be a key factor modulating the population of symbionts within the host.

## Introduction

For the last 200 million years, coral reefs have thrived in the shallow oligotrophic waters of tropical and subtropical oceans [1]. The success of scleractinian corals as reef builders during this period results from the metabolic advantages derived from the establishment of mutualistic symbioses with photosynthetic dinoflagellates in the genus *Symbiodinium* [2]–[3]. The translocation of photosynthates from the algal partner is essential to these symbioses, as in some instances it represents more than 100% of the basal respiratory requirements of the holosymbiont (animal + algae, sensu [4]) [5]–[7].

In general, glycerol is the main component of the newly fixed carbon translocated to the host, playing a key role in coral nutrition and growth [8]–[9]. Furthermore, the respiration of translocated glycerol may be the main energy source for coral calcification [10] while the resulting carbon is a significant component of the coral skeleton itself [11]–[12].

Despite its importance, very little is known about the mechanisms responsible for the production and release of low molecular weight photosynthates in the symbiosis. Carbon fixation and release from freshly isolated *Symbiodinium* cells occurs at very low rates, less than 5% of the measured fixed carbon [8], [13]–[14]. However, this can be induced by exposing freshly isolated *Symbiodinium* cells to host homogenate [8]–[9], [15]–[17] although the identity of the stimulating molecules is lost in the crude homogenate. Even a defined mixture of amino acids has been used as stimulus, although published results are reproducible under specific assay conditions and only in few instances with cultured *Symbiodinium* cells [13], [15], [18]–[20]. These facts have limited experimental studies dealing with the identity, production and translocation of photosynthates in the symbiotic algae.

Previous work published elsewhere has indicated that the type of photosynthates and the amount that is translocated to the host by symbiotic dinoflagellates varies among cnidarian species [14], [21]–[24]. However, the functional diversity displayed by *Symbiodinium* types has mainly been assessed by thermal tolerance and photosynthetic response, variables that are ecologically significant for coral bleaching ([e.g. [24] and references therein]). Such diversity is strongly associated with the fact that we are most probably dealing with different species within the genus *Symbiodinium*. We have included two types of *Symbiodinium* with different life histories: one symbiotic with the coral *Montastrea faveolata* (type B1) inhabiting mid depths in coral reefs, and the other with the medusa *Cassiopea xamachana* (type A1) inhabiting lagoons associated with mangroves. As an added advantage, there is some sequence information available for both *Symbiodinium* types (ESTs).

In many eukaryotic microorganisms, the synthesis of glycerol is induced by osmotic up-shocks. The best-known examples are halotolerant green alga in the genus *Dunaliella*, as well as almost all species of yeast studied to date [25]–[27]. By contrast, there is little information regarding the osmoregulatory capabilities of corals and their symbionts [28]. We have explored such conditions in order to induce the synthesis of glycerol in cultured *Symbiodinium*. Our main purpose was to generate an experimental setup that would allow the *in vitro* study of glycerol synthesis outside the host. Our results allowed us to suggest a mechanistic model for the synthesis and release of glycerol in *Symbiodinium* when *in hospitae*, that can also help explain the modulation of symbiont populations within the host.

## Results

### Short-term Effects of Osmotic Stress

#### Glycerol production

**Figure 1:**
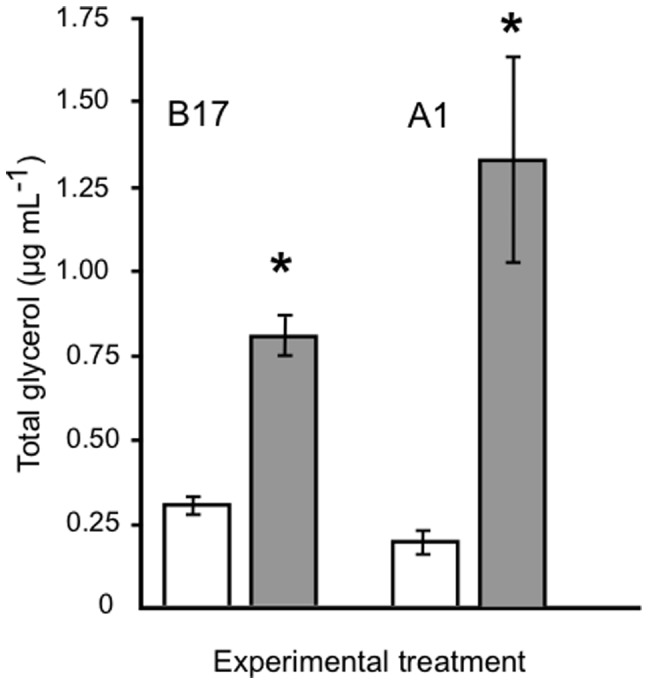
Experimental stimulation of glycerol production in *Symbiodinium*. Total glycerol (µg mL^−1^) produced by cultured *Symbiodinium* cells after 1 hr exposure to control conditions (white columns, minus symbol) or with 9% PEG up-shock (gray columns, plus symbol). Values adjusted to 1×10^5^ cells mL^−1^. *Symbiodinium* types indicated on top. Results show the average from 3 independent experiments ± SD (bars). Significant probabilities (P>0.05) for comparisons between treatments are shown with an asterisk.

In order to explore the possibility that glycerol release could be stimulated in Symbiodinium cells in culture, cells were challenged by osmotic up-shocks imposed by the addition of polyethylene glycol (since it is not taken up by the cultured cells). We employed a concentration of solute equivalent to 200 mM to study the response clearly. Total glycerol content was measured after 1 hr incubations under illuminated conditions. The assays were performed right after the end of the daily dark cycle in order to keep any carbon storages to a minimum. Results presented in Figure 1 indicate that glycerol was synthesized in response to osmotic up-shocks in both dinoflagellates studied. Total glycerol concentration significantly increased 2.5 and 5.3 times over control levels, for Symbiodinium type B17 and type A1, respectively. This response has also been observed with other solutes, when applied at equivalent osmolarities (Suescún-Bolívar et al, unpublished data). Controls were not significantly different but the response to the stress was significantly different between algal types (Figure 1).

Considering that the glycerol produced by the dinoflagellate cells could be ideally translocated to an animal host when in symbiosis, extracellular glycerol levels were estimated from cells incubated 1 hr under control or stress conditions. Results showed that most of the glycerol produced was detected in the extracellular medium, and only type A1 cells under control conditions had a significant difference between total and extracellular values (Table 1).

**Table 1:** Total and extracellular glycerol levels in *Symbiodinium* cells exposed to an osmotic up-shock.

	*Symbiodinium* type B17		*Symbiodinium* type A1	
	control	stressed	control	stressed
**Total**	127.39±10.54	252.94±76.73	140.05±10.94	218.25±22.30
**Extracellular**	130.51±9.36	191.08±16.03	106.65±4.28	208.52±15.97
**P**	0.790	0.239	**0.018**	0.354

Control cultures with no additions, stressed cultures with 9% PEG added. Cells were collected after 1 hr treatment. Glycerol concentration given in mg mL^−1^ in 10^5^ cells. Values are the means ± SD from three independent experiments. Significant probabilities (P) between control and stressed treatments for each cell type in bold (**<0.05**).

#### Photosynthesis

**Figure 2:**
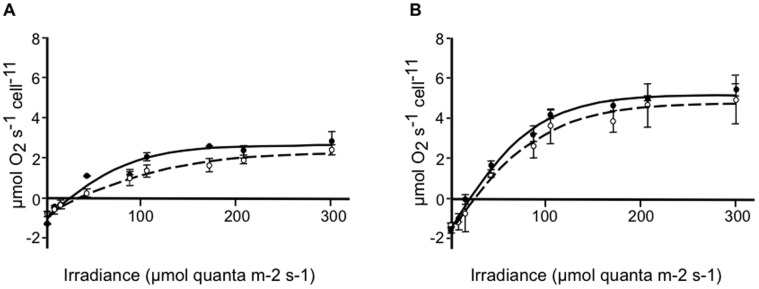
Photosynthesis *vs* irradiance (PE) curves normalized to cell density. Cells cultured for two weeks were incubated for 1 hr in control medium (solid line) or with the addition of 9% PEG (broken line). O_2_ evolution was assessed under increasing light intensity, in *Symbiodinium* type B17 (**A**) and type A1 (**B**) cells. Each value represents the mean ± SD (bars) from 3 independent experiments. Data were fitted to a hyperbolic tangent function.

Given the different life histories of the algal types used, and consistent with previous research [29], analyses of the photosynthetic responses under control conditions showed significant differences (Figure 2). However comparative analyzes of the parameters of the photosynthesis vs irradiance curves for control and osmotic stressed cultures indicated only minor differences (Table 2), although we detected significant reductions in the initial slope of the curve (α) for the osmotic stressed cultures of the B17 algae.

**Figure 3:**
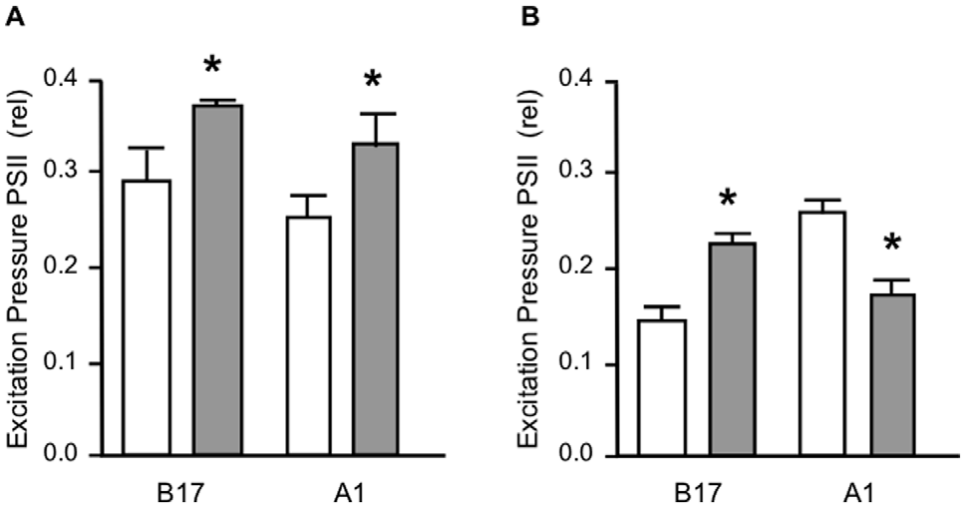
Excitation pressure over PSII. *Symbiodinium* cultures were exposed to high osmolarity conditions for (**A**) 1 hr or (**B**) 7 days, analyzed by fluorometry (see methods) and values for excitation pressure over PSII calculated for control conditions (white columns) or 9% PEG (gray columns). *Symbiodinium* types indicated on the ordinates. Results show the average from 3 independent experiments ± SD (bars).

Changes in the excitation pressure over photostystem II (Q) during the onset of osmotic stress, compared the quantum yields of charge separation (*Fv*/*Fm*) of dark “adapted” cells, with the *ΔF*/*Fm*’ of cultures exposed to supersaturating light intensities [30]. Q values stabilized at 30 min. At this time point stressed cultures of both algal types showed significant increases in the Q relative to controls (Figure 3). These results are consistent with the formation of a transient sink limitation at the end of the electron transport chain, where the increased demand of reducing power by ribulose 1,5-bisphosphate (Rubisco) associated with glycerol synthesis probably limits the electron flow towards other pathways such as nitrate reduction and assimilation [31].

**Table 2:** Photosynthetic parameters normalized to cell number for *Symbiodinium* cultured under experimental conditions.

	*Symbiodinium* type B17			*Symbiodinium* type A1		
Parameters	Control	Stressed	P	Control	Stressed	P
**P_max_**	1.70±0.274	1.44±0.148	0.838	3.24±0.175	3.07±0.642	0.938
**R**	0.76±0.292	0.48±0.076	0.207	0.95±0.077	0.79±0.049	0.793
**α** (10^−8^)	5.33±1.15	2.33±0.577	**0.016**	7.67±1.155	6.33±0.577	0.344

P_max_ and respiration (R) values expressed as µmol O_2_ min^−1^ cell^−9^. Samples are from two weeks old cultures after 1 hr incubations under control conditions or with the addition of 9% PEG (stress conditions). Values are means of 3 independent experiments ± SD. Significant probabilities (P) in bold (**<0.05**).

#### Expression of carbon fixation and glycerol synthesis genes

**Figure 4:**
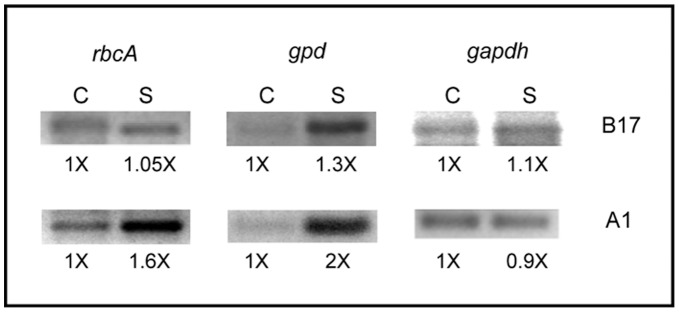
Expression of genes associated to carbon fixation (Rubisco, *rbcA*) and synthesis of glycerol (glycerol 3-phosphate dehydrogenase, *GPD*). RT-PCR from 1 µg RNA extracted from *Symbiodinium* type B17 (upper panel) or type A1 cultures (lower panel) exposed to control (left lanes) or osmotic stress conditions (right lanes) for 1 hr. The amplification products were separated in agarose gels photographed, scanned and quantified with the ImageJ program (Rasband, W.S., ImageJ, U. S. National Institutes of Health, Bethesda, Maryland, USA, http://imagej.nih.gov/ij/, 1997–2011). Color was inverted in the scans for clearer visualization. Numbers represent relative intensity of bands normalized to transcript levels for *GAPDH* (glyceraldehyde 3-phosphate dehydrogenase, see methods).

The expression of genes involved in carbon fixation and glycerol synthesis was analyzed by reverse transcription polymerase chain reaction (RT-PCR). Transcript levels were normalized to those measured for the housekeeping gene coding for glyceraldehyde 3-phosphate dehydrogenase (GAPDH). Rubisco enzyme catalyzes the rate-limiting step for carbon fixation in photosynthesis. Although a multigene family encodes this protein, the *rbcA* gene locus is predominantly expressed in peridinin-containing dinoflagellates [32]. Relative transcript levels for *rbcA* from cells cultured under control conditions were compared to cells exposed for 1 hr to osmotic up-shock. The expression of Rubisco in stress conditions in type B17 cells did not vary relative to the control, while in type A1 cells it increased approximately 1.6 times over the control (Figure 4). Glycerol 3-phosphate dehydrogenase (NAD(P)^+^-GPD), also known as dihydroxyacetone reductase, is an osmo-regulated enzyme in the synthesis pathway for glycerol [33]. The expression of this gene resulted also in higher transcript levels for the stressed cells, increasing approximately 1.3 times in type B17 and 2.0 times in type A1 over the expression levels in the controls (Figure 4). The expression pattern of these genes under osmotic stress conditions is in agreement with results presented earlier (Figure 1, Table 2).

### Long-term Effects of Osmotic Stress

#### Growth, glycerol production and photosynthesis

**Figure 5:**
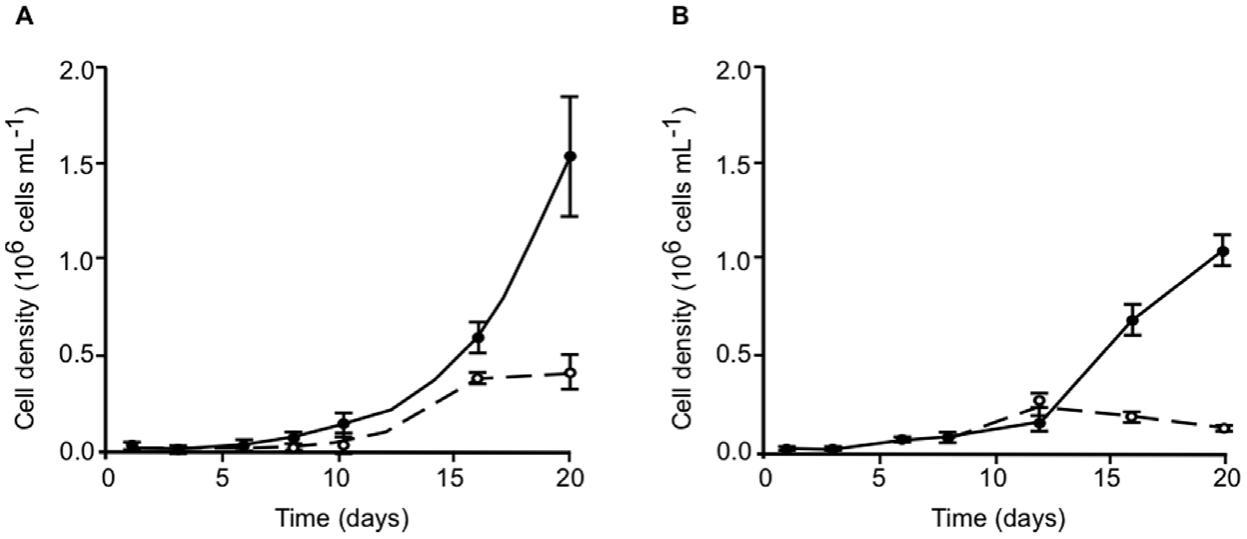
Growth of *Symbiodinium* cells under osmotic stress. (**A**) *Symbiodinium* type B17 and (**B**) type A1 cultures were inoculated with 1×10^4^ cells mL^−1^. After 6 days growing under control conditions (solid lines), half of the culture was transferred and stressed by adding 9% PEG (broken lines). Means ± SD from three independent experiments are shown.

The relation between the production of glycerol as a response to osmotic stress and growth was analyzed. *Symbiodinium* cells stressed by the addition of 9% PEG at the log phase of growth (6 days) showed a marked reduction in their division rate (Figure 5). We reasoned that the diversion of fixed carbon for glycerol production could be at least partially responsible for these results.

**Figure 6:**
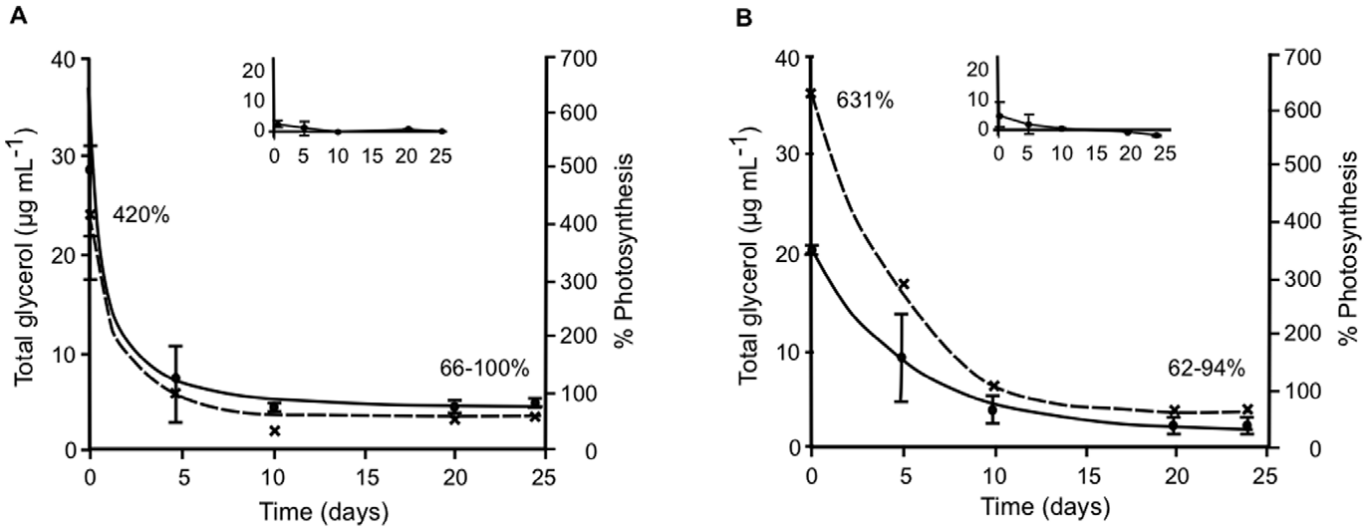
Percentage of photosynthesis needed to produce glycerol. Total glycerol (µg mL^−1^) was measured in *Symbiodinium* type B17 (**A**) and type A1 (**B**) cultured in medium with 9% PEG (solid line), sampled at the indicated times. Total glycerol values for control conditions are shown as an insert. Percentage of photosynthesis (broken line) estimated using P_max_ values (Tables 2 and 3) for the average glycerol concentration under stress at each time point. Values adjusted to 1×10^5^ cells mL^−1^. Mean values ± SD (bars) from three independent experiments.

Total glycerol contents of both *Symbiodinium* types grown under high osmotic conditions showed a significant increase in glycerol synthesis at the start of the experiments (Figure 6). Total glycerol decreased markedly and stabilized after 10 days, showing higher values than those estimated for cultures under control conditions (Figure 6). It is possible that the glycerol synthesized was partially replaced by other osmolytes in the course of the experiments. We ascertained if the synthesis of glycerol could be sustained by photosynthesis alone by converting oxygen evolution into fixed carbon. Photosynthesis alone would cover 66% (type A1) and 62% (type B17) of total glycerol production after 7 days of culture if we assume a photosynthetic quotient of 1, or 100% (type A1) and 94% (type B17) assuming a photosynthetic quotient of 0.6, consistent with equilibrated growth using nitrate as nitrogen source (Figure 6).

The photosynthetic performance of cultures grown for 7 days under high osmotic conditions normalized to cell content, showed a significant reduction in P_max_ in both algal types (Table 3). Photosynthetic parameters normalized to Chl *a* content replicated these results in type B17 cells, which also decreased significantly their dark respiration rates, indicative of a slower metabolism (Table 3). Chl *a* contents after 1 week of growth did not show appreciable variations in type B17 cells, while in type A1 cells Chl *a* was reduced to one half (Table 3) indicative of a photoacclimatory process.

The analysis of the excitation pressure over PSII for cultures grown 7 days under high osmolarity is presented in Figure 3. The excitation pressure for B17 type cells was again higher for stressed cells than for control cells 30 min after being exposed to light. However in A1 type cells the excitation pressure was reversed, being significantly higher now for control cells than for stressed cells. These results indicate that A1 type cells photoacclimated by increasing the number of electron sinks after one week of growth under high osmotic conditions, results that are in agreement with the reduction in both Chl *a* contents and P_max_ values normalized per cell (Table 3).

**Table 3:** Photosynthetic parameters for *Symbiodinium* cultures exposed to high osmolarity conditions for 7 days.

	*Symbiodinium* type B17			*Symbiodinium* type A1		
	CONTROL	STRESS	P	CONTROL	STRESS	P
**Normalized to cell number**						
**P_max_**	2.01±0.101	1.53±0.158	**0.014**	2.68±0. 147	1.54±0.144	**<0.001**
**R**	0.22±0.015	0.53±0. 065	0.534	1.10±0. 441	0.84±0.287	0.657
**α** ‡	3.00±0.00	3.00±0.00	1.00	5.33±2.08	5.67±1.15	0.245
**Chl ** ***a***	0.65±0.11	0.74±0.05	0.994	8.19±0.603	4.90±1.66	**0.009**
**Normalized to Chl ** ***a*** ** content**						
**P_max_**	6.24±0.31	4.17±0.429	**<0.001**	0.72±0.149	0.63±0.076	0.977
**R**	40.3±2.68	83.4±10	**<0.001**	15.6±6.29	20.0±6.8	0.869
**α** ‡‡	300.0±0.0	267±57.7	0.599	43.3±20.8	80.0±17.0	0.528

P_max_ and respiration (R) values expressed as µmol O_2_ min^−1^ cell^−9^ or µmol O_2_ mg^−1^ Chl *a* min^−1^ respectively. Chl *a* values expressed in mg mL^−1^. Values are the means of 3 independent experiments ± SD. Significant probabilities (P) in bold.

‡ **= **values×10^−8^;

‡‡ **= **values×10^−6^.

## Discussion

The mechanism that allows the production and translocation of glycerol in the symbioses between cnidarians and *Symbiodinium* algae has remained elusive since the phenomenon was first described [8]–[9], [34], [25]. Given the fact that glycerol is a molecule that functions as an osmolyte in many unicellular eukaryotes [35] and is a by-product in the metabolism of dinophytes [36], we experimentally explored inducing the synthesis of glycerol in response to an osmotic signal in *Symbiodinium*. We hypothesized that a signaling event following the same response pathway as an osmotic signal could provide insight on the mechanism underlying glycerol production when these cells are in symbiosis.

First, our results clearly demonstrate that symbiotic dinoflagellates in culture are able to produce glycerol given the appropriate conditions, as those employed here (e.g. osmotic stress), similar to what has been observed with freshly isolated algae exposed to host homogenate [8]–[9], [15]–[17], [37]. This suggests that *Symbiodinium* cells may employ the synthesis of glycerol as a rapid means to modulate their internal osmotic pressure, although in a long-term basis the internal osmotic pressure may be maintained by other, more stable organic molecules [38].

The prompt response to increase glycerol levels after a stimulus has been observed in freshly isolated symbionts exposed to host homogenate [17], [39], in the green alga *Dunaliella* [21], [33], [40]–[41], and has been amply observed in yeast cells incubated under similar conditions of high salt or sugar (see [22] for a review), reflecting the rapidity that characterizes what is termed the ‘glycerol response’ in yeast cells. In this respect, *Symbiodinium* seems to employ glycerol for the rapid adjustment of its internal osmotic pressure given a sudden change in external osmolarity. Although, as our results showed, *Symbiodinium* does not seem to be very osmo-tolerant since most of the glycerol produced is lost to the extracellular medium.

The demand for glycerol production after an osmotic stress forces a change in carbon allocation in the dinoflagellate, compromising growth of the cultured cells. This effect was apparent in our results, showing a decrease of division rates in cells that were exposed to high osmotic conditions. The growth rate decrease was not related to a diminished photosynthetic capacity, as short incubations after an osmotic up-shock had no significant effect on maximum photosynthesis (P_max_). Furthermore, fluorescence analyses indicated that the cultured cells perceived a higher pressure over PSII, while longer exposures to high osmolarity (7 days) did affect photosynthetic responses, particularly diminishing P_max_ per cell and eliciting a photoacclimatory response. In the highly salt-tolerant alga *Dunaliella tertiolecta* photosynthetic oxygen evolution increases by 30% when a mild salt stress is applied (e.g. from 0.17 M to 0.4 M NaCl), however above 0.7 M NaCl photosynthesis becomes inhibited and the same effect is observed with various salt and sugar solutes, indicative of a response to the osmotic pressure of the medium and possibly to the toxic effect of sodium [42]. However other algae like the fresh water *Chlamydomonas reinhardtii*, the euryhaline marine green alga *Chlorella autotrophica* and the flagellate *Chlamydomonas pulsatila*, diminish their carbon fixation rates when exposed to an osmotic stress but nevertheless are able to accumulate glycerol possibly employing carbon reserves [22], [40], [43].

Although glycerol has been detected in both the animal host and the algal partner in several symbiotic corals, low glycerol levels have been reported [38]. Nonetheless, glycerol was identified as the main photosynthate released from freshly isolated algae from symbiotic hosts [9]. The source of glycerol was clearly identified in *Fungia scutaria* larvae as it was detected only after infection with *Symbiodinium* [44]. Moreover, host homogenate from aposymbiotic *Anthopleura elegantissima* did not stimulate the release of glycerol from isolated algae, however this result was reversed after exposing isolated algae to host homogenate extracted from symbiotic anemone [15]. There are indications that glycerol is metabolized rapidly, which would explain that low levels are usually reported. For example, in the symbiotic anemone *Condylactis gigantea*, increased glycerol levels have been detected by partially uncoupling respiration from photosynthesis with sodium cyanide [16]. Our results with cultured *Symbiodinium* indicate that these algae are able to produce small amounts of glycerol not only after an osmotic up-shock but continuously, with the prevalence of high osmotic conditions.

The use of radioactive ^14^C carbon in experiments measuring newly fixed carbon in freshly isolated algae exposed to host homogenate, indicated that *Symbiodinium* converts about 50% of the newly fixed carbon into glycerol [9], [37], [39] employing little of the carbon reserves [45]–[46]. However, other studies had mixed results or were not able to measure increased carbon fixation rates upon exposure of the algae to host homogenate but only increased amounts of liberated carbon [46]–[47]. Our results showed that the amount of glycerol produced by an osmotic up-shock can be induced several fold over basal levels depending on the osmotic gradient established by the challenge. This carbon most probably comes from reserves, since the amount of carbon that would be available for glycerol production from P_max_ values indicates that glycerol synthesis takes up close to 100% of the newly fixed carbon. Even though we initiated all experiments right after the end of the daily dark cycle in order to keep carbon reserves to a minimum, reserves in the cultured algae were not fully depleted. In freshly isolated algae starch could be measured even after 16 hr incubations in the dark and a tendency for its decrease was only apparent after incubations of 4 hrs of cells exposed to host homogenate [46]. Our results showed the stabilization of glycerol production over time, indicating that glycerol after 10 days of culture is mainly produced from photosynthetic carbon fixation.

The synthesis of glycerol may impose a demand on photosynthesis such as to drive the flux of fixed carbon to glyceraldehyde 3-phosphate (GAPDH). The constant conversion of GAPDH into glycerol as the demand for this osmolyte is experimentally driven by the culture conditions, draws a fraction of the carbon flux away from the synthesis of structural and enzymatic molecules needed for growth, as well as from the recovery of Rubisco that allows the fixation of carbon. The consequence of this deviation as our results suggest, diminishes the efficiency of PSII, analogous to exposure to high irradiances, thermal stress or nutrient starvation [31], [48], increasing the expression of Rubisco.

A number of studies have been published concerning the release of photosynthetically fixed carbon from freshly isolated symbionts by exposing cells to host homogenate [8], [16]–[18]. However, *Symbiodinium* cells in culture could not be induced to produce appreciable amounts of glycerol by exposing them to host homogenate or a mixture of free amino acids (termed ‘synthetic host factor’), as occurs with freshly isolated algae [15], [18], [47] with only one exception [20]. This fact has limited experimental studies dealing with the production and true identity of photosynthates from the symbiont partner. Grant and collaborators [17] were able to determine that glycerol synthesis after incubating the symbiotic algae with host homogenate occurs only in the light, which could act as the driving force behind glycerol synthesis in the symbiosis. Our results indicate that cultured algae are able to use carbon reserves to respond to an osmotic challenge, but it has to be taken into consideration that we employed a steep osmotic gradient in our assays in order to clearly show its effects (equivalent to 200 mM of solute).

As our results suggest, the stimulation of glycerol synthesis can possibly reduce the division rates of *Symbiodinium* cells inside the host. Since photosynthesis should cover carbon demand, it may seem unreasonable to think of carbon as the limiting factor of symbionts cells *in hospitae*. However, a large fraction of the carbon fixed by the symbionts is lost (translocated) to the host and metabolized immediately [14], [16]. If carbon is lost in a continuous basis during daytime, the symbionts keep fixing carbon without covering their own quota for cell division, which translates in growth being modulated by carbon limitation although it may seem to be dependent on nitrogen availability, a hypothesis that has been challenged by several authors [31], [45]. Further, in cultures of *Symbiodinium* type A1 exposed to high osmolarity for several days, we observed a significant reduction of growth rates as well as a decrease of Chl *a* content, which could be interpreted as nutrient deficiency [7]. A more realistic interpretation would consider the lack of carbon skeletons to build proteins (with a C:N ratio of 3) due to the flux of carbon in the form of glycerol being lost from the cell [45].

As glycerol is the main photosynthate translocated to the animal host, our findings suggest a mechanism for its production in the symbiosis: by stimulating a putative osmosensor in the algal partner, the host could induce glycerol synthesis activating a signal transduction pathway through the osmotic response homologous to other eukaryotes [49]–[50]. For instance, in yeast cells one of three putative osmosensors located in the plasma membrane is activated in hypertonic conditions to initiate a signal transduction leading to glycerol synthesis through the HOG (High Osmolarity Glycerol) pathway [51]–[52]. Experimental evidence collected thus far indicates that this osmosensor interacts with other molecules in the cell wall for its function [53]–[55], which suggests that such osmosensor could be stimulated by a molecular interaction in the absence of an osmotic inducer. Although most studies have been performed with isolated algae, the identity of the photosynthate being translocated apparently varies with the symbiotic partners, and could further differ in the intact symbiosis [27], [56]–[57]. The implications that such variations could have in the holosymbionts are vast. To better understand the carbon flux that takes place in the diverse cnidarian-*Symbiodinium* symbioses, further research is required to address other possible photosynthates and assess the relative importance of glycerol.

### Concluding Remarks

Under the stimulating conditions for glycerol synthesis studied here, *Symbiodinium* cells had diminished growth and increased production of glycerol. Since photosynthetic parameters did not change significantly, the induction of Rubisco may have compensated for the higher demand for carbon fixation.

Our hypothesis does not suggest that an osmotic imbalance may be in effect in the intact symbiosis, but rather that a signaling event involving the osmotic response pathway may be responsible for the continued production of glycerol in the symbiosis. The proposed mechanism would have a double effect on the symbiosis, one being the production and translocation of glycerol that favors this relationship, the other a possible regulatory mechanism over the population of symbionts within the host.

## Materials and Methods

### *Symbiodinium* Cultures and Growth Conditions

Two symbiotic dinoflagellates with different genotypes were employed, *Symbiodinium sp.* ITS2 type A1 (isolate CassKB8 from the jellyfish *Cassiopeia* sp., Hawaii) and *Symbiodinium* sp. ITS2 type B17 (isolate Mf11.5b.1 from the coral *Montastrea faveolata*, Florida) from the culture collection of Dr. M. A. Coffroth and identified by Dr. Coffroth (SUNY at Buffalo, USA). Dinoflagellates were cultured in 300 mL flasks containing 100 mL of ASP-8A medium [58] at 24°C with fluorescent lights delivering 60 µmol quanta m^−2^ s^−1^ with a 12 hrs light-dark cycle. For osmotic up-shock experiments, 0.5 mL aliquots were incubated with solutes added from 2X concentrated stocks prepared in ASP-8A medium, in a 1 mL final volume. Experiments were initiated with 15 days-old cultures (exponential growth phase) grown under control conditions and sampled right after the end of the daily dark cycle. Cell density was adjusted to 1.2×10^7^ cells mL^−1^ by concentrating cultures by centrifugation. All experiments were repeated at least three times.

### Glycerol Estimation

Total glycerol was estimated after 1 hr incubations under experimental conditions, from 0.5 mL culture samples boiled for 15 min and clarified at 13,800×g for 10 min. For extracellular glycerol content, culture samples were centrifuged at 13,800×g for 5 min and the supernatant deproteinized by boiling for 10 min. Extracts were frozen to −70°C until analyzed. The glycerol content was estimated spectrophotometrically employing a colorimetric enzymatic assay (Free Glycerol Reagent A, SIGMA) and quantified against a glycerol standard. Estimations were repeated at least three times.

### Photosynthesis and Respiration Rates

Photosynthesis *vs* irradiance determinations were made in a water-jacketed chamber (DW1, Hansatech Instruments Ltd., UK) at 28°C following [29]. All measurements were made using cultures in exponential growth phase, sampled right after the daily dark cycle to keep accumulated reserves to a minimum. Cell density was set at 3.5×10^6^ cells in fresh medium with or without stressing solute (9% PEG). Respiration rates were first measured in cell suspensions by assessing O_2_ consumption in the dark. O_2_ levels were then depleted to 20% saturation by bubbling N_2_ gas. Cell suspensions were pre-incubated in the dark for 5 min, adding NaHCO_3_ to a final concentration of 5 mM to prevent CO_2_ limitation. Photosynthesis was then measured as the rate of net photosynthesis based on O_2_ evolution at increasing irradiance values, between 0 and 300 µmol quanta m^−2^ s^−1^, each applied for 3 min and monitored with a Clark-type electrode (Hansatech). Illumination was provided with a fiber optics illuminator (Cuda Products, USA). A hyperbolic tangent function was fitted to the data [59]. Photosynthetic parameters were standardized to cell density and chlorophyll *a* (Chl *a*) content. Chl *a* was extracted in cold acetone/dimethyl sulfoxide (90∶10, v/v) over night and quantified spectrophotometrically [60].

### Fluorescence Determinations

To assess the excitation pressure over photoystem II (PSII) as a measure of photochemical efficiency [30], fluorescence parameters were determined in cultures under the same conditions as those employed for photosynthesis *vs* irradiance curves. Maximum fluorescence of PSII was calculated from data collected with a pulse amplitude modulated fluorometer (diving PAM, Waltz, Germany). We employed an actinic light source with maximum output of 352 µmol quanta m^−1^ s^−2^ (CUDA 1–150) fitted with an attenuation filter. Cell density was set at 1.2×10^7^ cells mL^−1^ in an assay volume of 3 mL with or without PEG to a final concentration of 9%. Cell suspensions were pre-incubated for 1 hr in the dark to assure complete relaxation of PSII. Maximum quantum efficiency (*Fv*/*Fm*) and effective quantum efficiency (*ΔF*/*Fm*’) were measured in the assays after applying maximum intensity light pulses of 0.6 sec every 5 min during 50 min or until fluorescence values stabilized. The excitation pressure over PSII was determined by the relation Q = 1– [(*ΔF*/*Fm*’)/(*Fv*/*Fm*)] [30].

### RT-PCR of Rubisco and GPD Genes

Total RNA was extracted from cultures grown in control conditions or exposed for 1 hr to an osmotic up-shock (9% PEG), employing the Trizol reagent (Sigma) and a bead beater, following the recommendations by [61]. cDNAs were synthesized from 1 µg of total RNA using an oligo-dT_(15)_ primer with the ImProm II® Reverse Transcription System, according to the directions of the manufacturer (Promega, USA). The cDNA was PCR amplified employing specific primers for ribulose 1,5-bisphosphate carboxylase/oxigenase (Rubisco) form II, chosen from published sequences for the *rbcA* gene locus from *Symbiodinium* [32]. The amplification product was flanked by the oligonucelotide sequences 5′- TTCTCCGCCAACATAAC(t/c)GC-3′ (coding for amino acids FSANITA) upstream and 5′-AG(a/g)TTCTCGAAGAAGGC (c/t)GC-3′ (coding for amino acids PAFFENL) downstream, that amplify a region of 466 bp. For glycerol 3-phosphate dehydrogenase (NAD(P)^+^-GPD), or dihydroxyacetone phosphate reductase, the amplification was performed with primers chosen from amino acid sequences from dinofagellates, including a partial sequence from *Symbiodinium* type A1 with homology to GPDs. The primers had the sequence 5′-GTCCTCATGGGCGCCAAC-3′ (for amino acids VLMGAN) in the upstream primer and 5′- GGCGGCAGGAATCGAAAA-3′ (amino acids GGRNRK) for the downstream primer, amplifying a region of 378 bp.

In order to validate the relative changes in expression for the targeted genes, we measured RT-PCR transcript levels from the same cDNA samples, for the housekeeping gene glyceraldehyde 3-phosphate dehydrogenase (GAPDH), employing published primer sequences for the chloroplastic gene [62]–[63]. The RT-PCR products were separated in agarose gels stained with ethidium bromide, photographed, scanned and compared with the ImageJ program (Rasband, W.S., ImageJ, U. S. National Institutes of Health, Bethesda, Maryland, USA, http://imagej.nih.gov/ij/, 1997–2011). The relative abundances of GAPDH transcripts were for B17 type and 1X and 0.9X for A1 type, for control and stressed cells respectively. The small variation in GAPDH transcript levels was possibly due to total RNA or cDNA input. Transcript levels for the targeted genes were then normalized to these results.

### Growth of *Symbiodinium* Cultures under High Osmolarity

Experimental cultures under high osmotic stress were prepared by concentrating the cells to twice the desired density and adding 1 volume of 2X concentrated solute solutions made in ASP-8A medium. Control conditions were reproduced by the addition of culture medium only. For assessing the induction effect of high osmolarity on growth parameters, cultures were inoculated to a cell density of 1×10^4^ cells mL^−1^ and sampled every 2–3 days for cell numbers and total glycerol content. Conditions of high osmolarity were reproduced using polyethylene glycol (PEG, average MW 3,350) since it is not metabolized nor taken up by *Symbiodinium* cells. The final concentration used of 9% was equivalent to 200 mM for other sugars or 100 mM of NaCl, giving an estimated osmotic pressure of 1075 mOsm Kg^−1^ and producing clear results when compared to control conditions. Cell numbers were estimated by direct counting aliquots with a hemocytometer. A set of three cultures was monitored for each experimental condition.
